# Efficient and Accurate Synthesis for Array Pattern Shaping

**DOI:** 10.3390/s22155537

**Published:** 2022-07-25

**Authors:** Minseok Kang, Jaemin Baek

**Affiliations:** 1 Division of Electrical, Electronic, and Control Engineering, Kongju National University, Cheonan 31080, Korea; mskang@kongju.ac.kr; 2 Department of Mechanical Engineering, Gangneung-Wonju National University, Wonju 26403, Korea

**Keywords:** array pattern synthesis, mask template, phased arrays antenna, waveform pattern

## Abstract

Array pattern synthesis (APS) aims to create the desired array pattern as closely as possible to the prescribed mask template by varying the element excitations of the array. Herein, an efficient approach for the APS to control the sidelobe level is proposed. After designing the mask template to meet the prescribed sidelobe requirements and the waveform pattern, a set of element excitations is calculated through the Fourier transform performed on the projection the waveform pattern onto the mask template. Then, a desired array pattern can be synthesized from this updated set of excitation coefficients. The proposed APS approach directly presents a mathematical formulation of the exact set of excitations without any iterative optimization process. The proposed method is particularly suited for many array elements in linear antenna array. Thus, the proposed APS achieves substantial improvements in terms of computation complexity, performance, and ease of implementation in the algorithm when compared with conventional methods. Several simulation results are provided to verify the efficacy and effectiveness of the proposed method.

## 1. Introduction

The use of array pattern synthesis (APS) in applications can be maximized through several essential factors, including digital beamforming, array pattern optimization, and subarrays. An array pattern can electronically scan array beams in space even if the array does not physically move [1,2,3,4]. The phased-arrays can exhibit arbitrary APS characteristics, which are based on an adequate control of sidelobe level (SLL), by varying the amplitude and phase excitations for each element [5,6,7,8]. Generally, element currents are iteratively investigated through the optimization process to reduce the error between the desired APS and the prescribed mask template [9,10,11,12].

To address the APS problem, numerous methods have been devised in the literatures [13,14,15,16,17,18,19,20,21,22,23]. The APS optimization problem is addressed by many effective approaches, such as non-convex (namely stochastic optimizer) optimization, convex optimization, and sparse recovery method. Non-convex optimization techniques based on nature-inspired algorithms (e.g., stochastic optimizers such as particle swarm optimization (PSO) [9], genetic algorithms [13], differential evolution algorithm [14] and ant colony optimization algorithm [15]) adjust the trial solutions and attempt to converge to the global optimum by repeatedly evaluating the cost function with different trial solutions. The resultant optimized patterns for these optimizers perfectly fit the mask template. However, stochastic optimization methods are associated with huge amounts of computational complexity, since the required number of synthesis iterations considerably increases depending on the size of the array [24,25,26]. Meanwhile, convex optimization techniques, such as quadratic error minimization and linear programming (LP), is successfully applied in handling the issue of APS optimization problem [16,17]. An analytical technique proposed in [16] is efficient for solving the problem by means of LP procedure followed by a polynomial factorization. Furthermore, the authors extended a preliminary version of theory and procedures in [16] for the case where an even distribution is required on element excitations [17]. In [18], the author utilizes successive fast-Fourier transform (FFT) to produce the thinned APS. This method achieves improvements in terms of computation complexity and ease of implementation in the algorithm. However, the method suffers from drawbacks such as sensitivity to the selection of the initial point, which results in a huge number of iterations to acquire the desired results. The efficient APS approaches based on the iterative convex optimization are introduced in [19,20]. However, the interior point method requires high computational complexity to solve convex optimization related to APS tasks with large-sized array [21]. Likewise, the large-sized array makes it challenging to provide efficient APS approaches using the matrix enhancement and matrix pencil synthesis method proposed in [22], due to the requirement of a huge amount of computing resources and memory. In [23], the authors present a novel APS scheme using a fast iterative shrinkage-thresholding algorithm (FISTA) based on sparse optimization. However, in some cases, this approach fails to find the global minimum corresponding to the desired APS due to the cost surface being trapped in local minima when dealing with large-sized arrays [27].

Motivated by the problems of the conventional optimization methods [13,14,15,16,17,18,19,20,21,22,23], a completely different approach for the APS is presented in this paper. The proposed scheme is composed of three steps: (1) design of arbitrary mask template and waveform pattern, (2) discrete Fourier transform (DFT) process of the projection of the waveform pattern onto the mask template, and (3) the calculation of the desired *AF* using the updated set of element excitations. The proposed APS approach directly provides a mathematical formulation of the exact set of element excitations, which yield the desired APS without any iterative optimization process. Since the core calculations in the proposed approach rely on the fast-Fourier transform (FFT) operations, the proposed method is computationally more efficient than the traditional optimization process, essentially coming down to trial and error. Furthermore, the proposed method is especially suited for many array elements in linear antenna array.

The organization of this paper is as follows: In Section 2, we introduce the problem statement of APS. In Section 3, a mathematical framework for the proposed method is derived in detail. The results of several simulations are provided to demonstrate the proposed scheme in Section 4. Section 5 discusses the results in perspective of previous studies, highlighting certain future research directions. Finally, several conclusions are presented in Section 6.

## 2. Problem Formulation and Brief Description of Proposed Method

The array factor (*AF*) resulting from an array of identical discrete elements is the sum of the radiations for each element excited by the spatial phase delay from each element to the far-field point. The *AF* sum can be obtained in an approach that is similar to a Fourier series. The desired function of AF(u) can be represented as a Fourier series in the interval −d/λ<u<d/λ as [6]

(1) $$AF(u)=\sum_{i=-\infty }^{\infty }c_{i}e^{j2\pi i\frac{d}{\lambda }u}$$

(2) $$c_{i}=\frac{d}{\lambda }\int_{-\frac{\lambda }{2d}}^{\frac{\lambda }{2d}}AF(u)e^{-j2\pi i\frac{d}{\lambda }u}du$$

where λ is the wavelength. It is assumed that the elements are uniformly spaced at distance *d* and u=cosθ, where θ is the angle from the line of the linear array. The summation of (1) is recognized as the *AF* of an array with an infinite number of elements with currents $c_{i}$, called as *i*-th element excitation. The AF(u) arising from these element currents approximates the desired function. The complete pattern representation for the array is found using pattern multiplication. The pattern multiplication means that the complete pattern is calculated by multiplying the element pattern E(u) and AF(u). In (3), it is shown the total pattern P(u) for the array of element excitations:

(3) P(u)=E(u)×AF(u)

It is generally assumed that E(u) is identical for each element in the electronically scanned arrays when the mutual coupling effect is negligible.

In this paper, we focus on the estimation of a set of amplitudes and phases of the excitation source $c_{i}$ to synthesize the desired AF(u) by exploiting the direct solution approach, rather than solving the optimization problem related to APS. Then, the desired AF(u) as closely as possible to the prescribed mask template can be obtained from the superposition of the element pattern from an updated set of element excitations. A brief description of the proposed beamforming approach based on the mask template and waveform pattern is provided below. Consider a linear array of *K* elements with an array of length (*K*−1)*d*. The mask template vector $m_{\mathrm{dB}}$ designed to meet the prescribed sidelobe requirements in decibel scale is transformed to linear scale data $m=(m_{1},m_{2},\cdots ,m_{K})\in {\mathbb{R}}^{K}$ for implementing the proposed method. The desired *AF* can be represented as

(4) a=m⊙w

where ⊙ denotes the Hadamard operator (entrywise product), $a=(AF_{1},AF_{2},\cdots ,AF_{K})\in {\mathbb{R}}^{K}$ is the desired *AF*, which can be interpreted as the projection of the vector w whose entries $(w_{1},w_{2},\cdots ,w_{K})\in {\mathbb{R}}^{K}$ consist of the designed waveform in *K* sampling points onto the mask template m discretized in the same positions. Furthermore, a can also be rewritten as the product of the inverse DFT matrix and the excitation source vector $c=(c_{1},c_{2},\cdots ,c_{K})\in {\mathbb{C}}^{K}$:

(5) $$a=F^{H}c$$

where $F^{H}$ is the Hermitian of $F\in {\mathbb{C}}^{K\times K}$ DFT matrix with entries $F_{n,k}=1/\sqrt{K}e^{-j2\pi nk/K}$ and denotes the inverse DFT. Therefore, from (4) and (5), we can obtain the following relation:

(6) $$\hat{c}=F\left[m⊙w\right].$$

Based on the descriptions in (4)–(6), an updated set of element excitations $\hat{c}$ can yield the desired *AF*, which fully matches the SLL requirements related to the prescribed mask template. The proposed beamforming method based on the efficient and robust approach allows extremely fast excitation source estimation by significantly reducing the computational load. The mathematical framework for the proposed method is derived in detail in the following paragraph.

## 3. Mathematical Formulation of Efficient Approach to Array Pattern Synthesis

The proposed beamforming method can be formulated with the help of mask template design and waveform pattern. The mask template is comprised of the rectangular functions as a one of the families of orthogonal functions used to form a basis. Thus, any mask template could be written as a linear combination of the rectangular function:

(7) $$\begin{array}{cc} m(u) & =\sum_{n=0}^{L-1}b_{n}\Pi \left(\frac{u-u_{n}}{\tau_{n}}\right)\\ & =b_{M}\Pi \left(\frac{u-u_{M}}{\tau_{M}}\right)+\sum_{\begin{array}{l} n=0\\ n\neq M \end{array}}^{L-1}b_{n}\Pi \left(\frac{u-u_{n}}{\tau_{n}}\right), \end{array}$$

where *L* represents the total number of rectangular functions, Π(⋅) denotes the rectangular function, $b_{n}$ is the scaling constant of the *n*-th rectangular function. Further, $u_{n}$ and $\tau_{n}$ are a time-delay and width of the *n*-th rectangular, respectively. Similar to the description of the *n*-th rectangular, $b_{M}$, $u_{M}$, and $\tau_{M}$ are defined in the *M*-th rectangular function corresponding to the mainlobe.

To design the waveform pattern, a one-to-one correspondence between the mask template and the waveform pattern should be established in sine space (−1<u<1). Furthermore, zero-crossing points of sinc function should be calculated with respect to the initial beam pattern, since it is imperative that the waveform pattern has the same zero-crossing points without the attenuation of the sinc function. The waveform pattern w(u) can be expressed as:

(8) $$w(u)=\{\begin{array}{l} \cos(2\pi \alpha u),\;|u|\leq \frac{1}{4\alpha }\\ \sin\left\{2\pi (2\alpha )u\right\},\;|u|>\frac{1}{4\alpha } \end{array},$$

where α denotes the parameter to be determined by the mainlobe width (considering the presence of a large number of array elements) of the mask template. The first zero-crossing point of sinc function is calculated at u=1/(4α) when cos(2παu)=0 for 2παu=π/2. Then, $\alpha =1/(2\tau_{M})$ can be estimated from the mathematical relation $\tau_{M}=2u$ between the mask template and waveform pattern with respect to the mainlobe. The rest of the zero-crossing points in the sidelobe region of sinc function are located in positions that are based by sin{2π(2α)u}=0. In this study, we adopted $\alpha =1/\left[2\tau_{M}\times (1+\delta )\right]$, where δ=0.1, considering the practical design specifications and constraints. Using the rectangular function, (8) can be rewritten as

(9) $$w(u)=\Pi \left(\frac{u-u_{M}}{\tau_{M}}\right)\cos(2\pi \alpha u)+\left[1-\Pi \left(\frac{u-u_{M}}{\tau_{M}}\right)\right]\sin\left\{2\pi (2\alpha )u\right\}.$$

The desired *AF* can be written as the multiple of m(u) and w(u):

(10) $$\begin{array}{c} \begin{array}{cc} p(u) & =m(u)\times w(u)\\ & =\left[\underset{mainlobe\;of\;m(u):\;m_{m}(u)}{\underset{︸}{b_{M}\Pi \left(\frac{u-u_{M}}{\tau_{M}}\right)}}+\underset{sidelobe\;of\;m(u):\;m_{s}(u)}{\underset{︸}{\sum_{\begin{array}{l} n=0\\ n\neq M \end{array}}^{L-1}b_{n}\Pi \left(\frac{u-u_{n}}{\tau_{n}}\right)}}\right] \end{array}\\ \times [\underset{mainlobe\;of\;w(u):\;w_{m}(u)}{\underset{︸}{\Pi \left(\frac{u-u_{M}}{\tau_{M}}\right)\cos(2\pi \alpha u)}}+\underset{sidelobe\;of\;w(u):\;w_{s}(u)}{\underset{︸}{\left[1-\Pi \left(\frac{u-u_{M}}{\tau_{M}}\right)\right]\sin\left\{2\pi (2\alpha )u\right\}}}]\\ =[\underset{mainlobe\;of\;p(u):\;p_{m}(u)}{\underset{︸}{b_{M}\Pi \left(\frac{u-u_{M}}{\tau_{M}}\right)\cos(2\pi \alpha u)}}+\underset{sidelobe\;of\;p(u):\;p_{s}(u)}{\underset{︸}{\sum_{\begin{array}{l} n=0\\ n\neq M \end{array}}^{L-1}b_{n}\Pi \left(\frac{u-u_{n}}{\tau_{n}}\right)\left[1-\Pi \left(\frac{u-u_{M}}{\tau_{M}}\right)\right]\sin\left\{2\pi (2\alpha )u\right\}}}], \end{array}$$

where $p_{m}(u)=m_{m}(u)\times w_{m}(u)$ and $p_{s}(u)=m_{s}(u)\times w_{s}(u)$. $p_{m}(u)$ of the last equality in (10) is derived by $\Pi^{2}\left[\left(u-u_{M}\right)/\tau_{M}\right]=\Pi \left[\left(u-u_{M}\right)/\tau_{M}\right]$. The beam pattern p(u) is composed of two main parts: the mainlobe part $p_{m}(u)$ and the sidelobe part $p_{s}(u)$.

The frequency response $P_{m}(f)$ can be generated with a Fourier transform (*FT*) of $p_{m}(u)$ with respect to variable *u*, as follows:

(11) $$\begin{array}{cc} P_{m}(f) & =FT_{u}\left[p_{m}(u)\right]\\ & =b_{M}\tau_{M}\sin c(f\tau_{M})e^{-j2\pi fu_{M}}{⊗}_{f}\left[\frac{\delta (f-\alpha )+\delta (f+\alpha )}{2}\right], \end{array}$$

where ${⊗}_{f}$ denotes the convolution operation over the frequency *f* sinc(⋅) and δ(⋅) are the sinc and the Dirac delta functions, respectively. Based on the notion that a set of rectangular function in an inner product space is an orthogonal set, $p_{s}(u)$ can be rewritten as:

(12) $$\begin{array}{c} p_{s}(u)=\sum_{n=0}^{L-1}b_{n}\Pi \left(\frac{u-u_{n}}{\tau_{n}}\right)\left[1-\Pi \left(\frac{u-u_{M}}{\tau_{M}}\right)\right]\sin\left\{2\pi (2\alpha )u\right\}-b_{M}\Pi \left(\frac{u-u_{M}}{\tau_{M}}\right)\left[1-\Pi \left(\frac{u-u_{M}}{\tau_{M}}\right)\right]\sin\left\{2\pi (2\alpha )u\right\}\\ =\sum_{n=0}^{L-1}b_{n}\Pi \left(\frac{u-u_{n}}{\tau_{n}}\right)\left[1-\Pi \left(\frac{u-u_{M}}{\tau_{M}}\right)\right]\sin\left\{2\pi (2\alpha )u\right\}\\ =\sum_{n=0}^{L-1}b_{n}\Pi \left(\frac{u-u_{n}}{\tau_{n}}\right)\sin\left\{2\pi (2\alpha )u\right\}-b_{M}\Pi \left(\frac{u-u_{M}}{\tau_{M}}\right)\sin\left\{2\pi (2\alpha )u\right\}. \end{array}$$

$\sum_{n=0}^{L-1}\Pi \left[\left(u-u_{n}\right)/\tau_{n}\right]\times \Pi \left[\left(u-u_{M}\right)/\tau_{M}\right]=\Pi \left[\left(u-u_{M}\right)/\tau_{M}\right]$ is applied in last equality as the orthogonal property in (12). Then, the frequency response of $p_{s}(u)$ can be obtained by the *FT* of $p_{s}(t)$ along the time direction, as follows:

(13) $$\begin{array}{c} \begin{array}{cc} P_{s}(f) & =FT_{t}\left[p_{s}(u)\right]\\ & =\sum_{n=0}^{L-1}b_{n}\tau_{n}\sin c(f\tau_{n})e^{-j2\pi fu_{n}}{⊗}_{f}\left[e^{j\frac{\pi }{2}}\frac{\delta (f+2\alpha )-\delta (f-2\alpha )}{2}\right] \end{array}\\ +b_{M}\tau_{M}\sin c(f\tau_{M})e^{-j2\pi fu_{M}}{⊗}_{f}\left[e^{j\frac{\pi }{2}}\frac{\delta (f-2\alpha )-\delta (f+2\alpha )}{2}\right]. \end{array}$$

Note that information about |c| and ∠c can be easily obtained by carrying out a *FT* over p(t) along the time direction as follows:

(14) $$\begin{array}{cc} P(f) & =FT_{t}\left[p(u)\right]\\ & =FT_{u}\left[\left\{m_{m}(u)+m_{s}(u)\right\}\times \left\{w_{m}(u)+w_{s}(u)\right\}\right]\\ & =FT_{u}\left[p_{m}(u)\right]{⊗}_{f}FT_{u}\left[p_{s}(u)\right]\\ & =P_{m}(f){⊗}_{f}P_{s}(f). \end{array}$$

An inequality $\left|P_{m}(f)\right|\geq \left|P_{s}(f)\right|$ holds true because the dominant sinc function ($b_{M}\tau_{M}\gg b_{n}\tau_{n}$) related to the mainlobe pattern corresponding to the *M*-th rectangular function ($b_{M}\gg b_{n}$), is always guaranteed. Thus, |c|=|P(f)| and ∠c=∠P(f) can be respectively approximated by

(15) $$\left|P(f)\right|≃\left|P_{m}(f)\right|$$

(16) $$∠P(f)≃∠P_{m}(f).$$

From (11) and (13), we can observe that the spectrum |P(f)| is roughly close to a single dominant sinc, which is induced by the superposition of two sinc functions separated by 2α in (11). Likewise, ∠P(f) is similar to the wrapped phase measurements, which consists of a combination of two linear functions.

**Figure 1:**
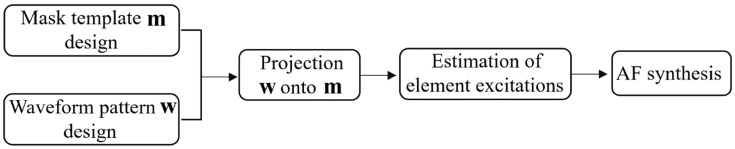
Overall flowchart of the proposed approach for APS.

Finally, the desired *AF* can be directly approximated calculating (1) using the excitation sources determined in (14). The overall flowchart of the proposed framework for the efficient approach to APS is presented in Figure 1.

## 4. Experimental Results

In this section, some examples are presented to verify the effectiveness of the proposed APS approach in terms of performance improvement and computational efficiency. All computations were run by MATLAB in Windows 10 on an AMD Ryzen 9 5900X 12-Core Processor at 3.70 GHz.

### 4.1. Simulation Results of APS Using Proposed Approach

**Figure 2:**
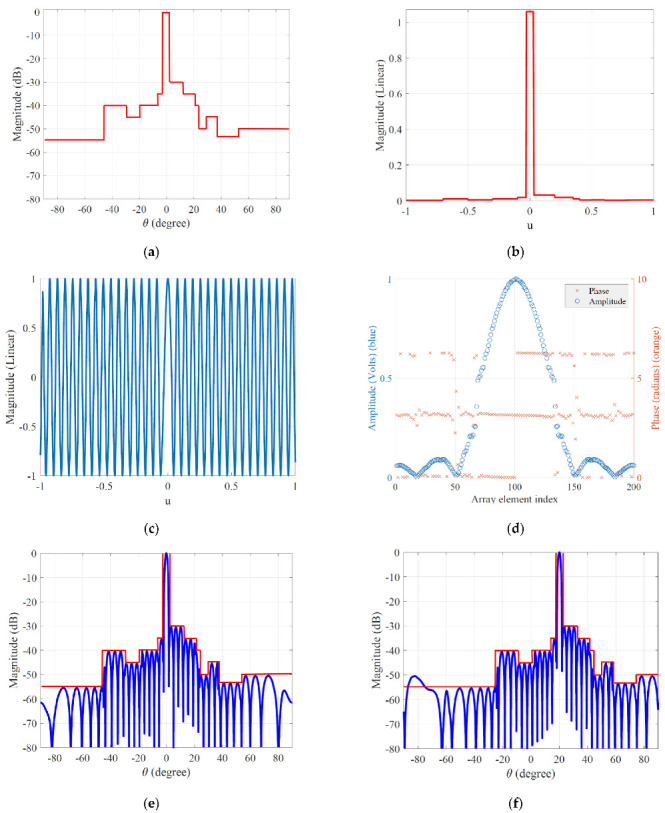
(**a**) Designed mask template in dB scale; (**b**) Designed mask template in linear scale; (**c**) Designed waveform pattern; (**d**) A set of element excitations calculated using (**c**,**b**) with respect to both amplitude (blue) and phase (orange); (**e**) The desired AF synthesized by a set of element ex-citations of (**d**); (**f**) The desired *AF* synthesis steered towards 20 degrees in the presence of defec-tive elements.

In this subsection, we verify the performance of the proposed technique for APS with a linear array. The DFT function is used to evaluate the far-field pattern, which implicitly assumes that the phased array elements are spaced at half wavelength. Thus, the case of a linear array with *K* = 200, λ/2-spaced isotropic antennas is considered. As shown in Figure 2a, the mask template $m_{\mathrm{dB}}$ is designed such that it suffices the prescribed SLL requirements for the desired APS beforehand, by using a linear combination of the rectangular function. Thus, m can be obtained by executing a linear scaling of $m_{\mathrm{dB}}$ (Figure 2b). The waveform pattern w can be constructed with the information associated with the beamwidth $\tau_{M}$ and the calculation of zero-crossing point of initial beam pattern having a uniform distribution (Figure 2c). A set of element excitations P(f) (both amplitude and phase) is calculated through the DFT, which is performed on the projection w onto m. As expected, it is clearly observed in Figure 2d that most energies of P(f) are concentrated at the center of the array distribution, because two main sinc functions of (11) are closely aligned. Finally, Figure 2e shows that numerical vectors m and w, which are designed using the proposed method, are converted to the desired *AF* against c. The directivity of the APS attained by the proposed method has the value of 30.94 dB. Furthermore, for a more realistic evaluation [28] of the proposed method, antenna pattern recovery with beam steering simulation is performed for 5% randomly dispersed element failure to analyze the pattern generation performance of the proposed method. The defective elements are randomly chosen over the linear array, and their excitations are set to zero in order to simulate the defective elements. In the presence of defective elements, the desired beam steering, which is about changing the direction of the mainlobe of a radiation pattern, is obtained by the proposed method, as shown in Figure 2f. While the APS with a directivity of 30.72 dB successfully steered toward 20 degrees, the excess SLLs outside the upper bounds are observed to increase, as shown in Figure 2f. The proposed method does not rely on a cost function to solve the constrained optimization problem or the adjustment of parameter to implement adequate control of SLLs and is thus very straightforward to use. This indicated that the proposed beamforming method based on the efficiency of the FFT, capable of providing correctly designed and desired APS, allows extremely fast and accurate excitation source estimation by significantly reducing the computational load. 

### 4.2. Comparison of Performance Analysis for APS Techniques

**Figure 3:**
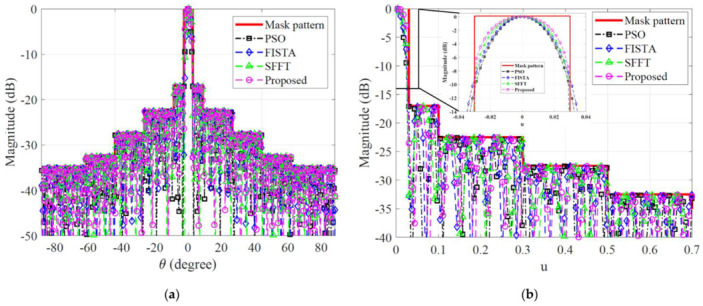
Desired AFs synthesized with *K* = 300 array elements for desired beamwidth $\tau_{M}=0.06$ (rad), and the comparison between the proposed method and optimization techniques; (**a**) Full angle range; (**b**) Sector beam patterns.

Some examples are presented to compare the performance of the proposed APS approach to that of well-established state of the art methods, such as the successive FFT (SFFT) [18], FISTA [23], and PSO [9] algorithms based on each of the three different types of optimization approaches. All optimizers generate trial solutions as a set of numbers between 0 and 1, which are then linearly mapped to the amplitude and phase at each element. The representative examples are presented to illustrate how each algorithm achieves such different results in dealing with the same APS task in terms of synthesis accuracy and computational efficiency. The resultant patterns optimized by each APS algorithms are well-formed to fit the mask template and are almost analogous within the full angle range in Figure 3a. The directivity of the APS attained by the FISTA has the smallest value of 32.69 dB among the four algorithms. On the contrary, the directivity measured by the proposed method (32.78 dB) is better than the SFFT (32.76 dB) and PSO (32.73 dB). Figure 3b shows that a substantial flatness is achieved in the mainlobe area in the proposed method and the radiation in the transition area is sufficiently suppressed, which is in contrast to the other methods. The *AF* of the proposed method decreases in its transition region much more rapidly than those of other methods. Furthermore, the angular span between the first pattern nulls adjacent to the mainlobe within its transition area provided by the proposed method, which is 0.059 rad, and is the smallest among all radiation patterns. The *AF* of the proposed method decreases in its transition region more rapidly than those of other methods. Furthermore, the angular span between the first pattern nulls adjacent to the mainlobe within its transition area, provided by the proposed method, which is 0.059, and is the smallest among all radiation patterns.

**Figure 4:**
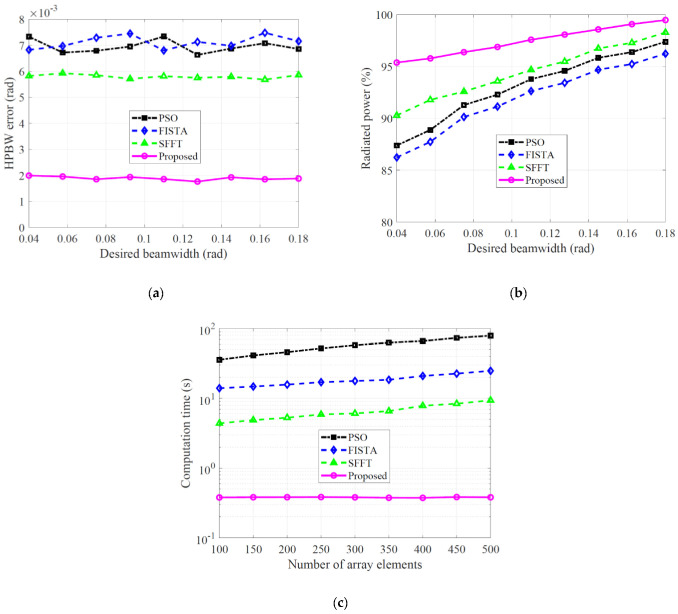
(**a**) HPBW error calculated as the absolute value of the difference between synthesized and desired HPBWs; (**b**) Percentage of power radiated by each APS technique in the mainlobe region; (**c**) Average computation times (on a semilogarithmic scale) required to synthesize desired *AF* with beamwidth $\tau_{M}=0.07$ (rad) versus the number of array elements for each APS technique.

To verify the effectiveness of the proposed approach in terms of the ability to generate the desired APS to be form-fit into a mask template, we calculated the half-power beamwidth (HPBW) error as the absolute value of the difference between the HPBW of the APS and the desired HPBW, as varying the desired beamwidth. Observations of Figure 4a showed that the HPBW error of the resultant APS attained by the proposed method was below 0.002 rad over all the metrics, which was approximately 67%, 70%, and 71% lower compared to that of SFFT, PSO, and FISTA, respectively.

Meanwhile, we also analyzed the percentage of power radiated by the APS in the mainlobe region described in Figure 3b. The numerical results were measured by the radiated power of the desired *AF* synthesis obtained using each algorithm. The radiated power of the four algorithms gradually increases in proportion to the value of desired beamwidth, as shown in Figure 4b. However, the proposed APS leads to the best performance among all radiation patterns over the range of all desired beamwidth. Thus, it can be concluded that the proposed method considerably outperforms the other methods in terms of the ability to concentrate the radiated power in the mainlobe region, regardless of the desired beamwidth.

Further analysis of the *AF* synthesis was considered to investigate the performance of the proposed method for a large number of array excitation elements. The numerical results were analyzed by using the computational complexity required by each algorithm in order to provide the *AF* synthesis with a desired beamwidth $\tau_{M}=0.07$ rad. All methods were performed under different *K* in the range from 100 to 500 with a 50 step, in which 50 Monte-Carlo simulations were conducted at each *K* (the number of array elements). The average computation time for each of the four APS methods were displayed in Figure 4c. It was observed that the proposed method conducted the search for an optimal solution with faster estimation than four different APS methods. The computation time of the three algorithms gradually increased with the number of array elements. However, it was worth noting that the computation time of the proposed method was considerably reduced and was relatively unaffected by the number of array elements. The proposed APS approach is especially appropriate for a large number of array excitation elements. Therefore, we can conclude that the proposed method can considerably improve the computational efficiency, while retaining the synthesis quality of the desired far-field pattern, compared to SFFT, FISTA and PSO.

## 5. Discussion

An attractive attribute of the proposed method is that the proposed beamforming method based on the efficient and robust approach allows extremely fast excitation source estimation by significantly reducing the computational load. An updated set of element excitations can yield the desired *AF*, which fully matches the SLL requirements related to the prescribed mask template. The mathematical framework for the proposed method is derived in detail. The proposed scheme is composed of three steps: (1) design of an arbitrary mask template and waveform pattern, (2) DFT process of the projection the waveform pattern onto the mask template, and (3) the calculation of the desired *AF* using the updated set of element excitations. The proposed APS approach directly provides a mathematical formulation of the exact set of element excitations, which yield the desired APS without any iterative optimization process. Since the core calculations in the proposed approach rely on the FFT operations [29,30,31,32,33,34], the proposed method is computationally more efficient than the traditional optimization process, essentially coming down to trial and error [35,36,37,38,39]. Generally, it is assumed that the elements of linear array are uniformly spaced at distance *d*. Thus, the proposed APS has been developed to achieve satisfactory performance from fully and uniformly sampled data. The effective use of proposed APS can address an important issue [40,41,42,43] in the framework of radar missions [44,45,46] because the proposed method has the ability to create almost arbitrary APS characteristics based on adequate control of SLL. Furthermore, the proposed method is especially appropriate for the synthesis of large-sized linear arrays such as pencil beam [47,48] because the complicated design of mask template requires a large number of arrays, namely the wide bandwidth. Therefore, we can conclude that the proposed method can considerably improve the computational efficiency, while retaining the synthesis quality of the desired far-field pattern, compared to conventional APS methods. On the other hand, the proposed APS approach suffers from certain limitations, such as null placement [49] in APS and difficulty in the optimization of directivity [50], because the proposed method focuses on managing the task to exhibit arbitrary APS characteristics, which are based on an adequate control of SLL, by varying the amplitude and phase excitations for each element. Thus, the future work will be devoted to extending the proposed APS to null placement and optimization of directivity task.

## 6. Conclusions

In this study, the efficient APS approach of linear arrays with a periodic arrangement of the elements is devised. The proposed approach entails following steps: (1) the design of the prescribed mask template m and waveform pattern w, (2) the DFT process of the projection w onto m in the sampling positions, and (3) the calculation of the desired *AF* from the estimated set of array elements c. In the simulation results, the proposed scheme demonstrates an excellent performance of accurate APS to meet the prescribed sidelobe requirements and outperforms significantly better in terms of efficiency, robustness, and ease of use in the algorithm when compared to the conventional methods. The proposed approach is particularly appropriate for the synthesis of large-sized linear arrays since all the excitation coefficients can be estimated simultaneously through FFT operation.

## Data Availability

Not applicable.
